# A small population, randomised, placebo-controlled trial to determine the efficacy of anakinra in the treatment of pustular psoriasis: study protocol for the APRICOT trial

**DOI:** 10.1186/s13063-018-2841-y

**Published:** 2018-08-29

**Authors:** Victoria Cornelius, Rosemary Wilson, Suzie Cro, Jonathan Barker, David Burden, Christopher E. M. Griffiths, Helen Lachmann, Helen McAteer, Nick Reynolds, Andrew Pink, Richard B. Warren, Francesca Capon, Catherine Smith

**Affiliations:** 1 Imperial Clinical Trials Unit, School of Public Health, Imperial College London, Stadium House, 68 Wood Lane, London, W12 7RH UK; 2 St John’s Institute of Dermatology, Guy’s and St Thomas’ NHS Foundation Trust, 9th Floor Tower Wing, Guy’s Hospital, London, UK; 3 St John’s Institute of Dermatology, Faculty of Life Sciences and Medicine, King’s College London, London, UK; 4 Department of Dermatology, Royal Infirmary, Edinburgh, UK; 5 The Dermatology Centre, Salford Royal NHS Foundation Trust, University of Manchester, Manchester Academic Health Science Centre, Manchester, UK; 6 National Amyloidosis Centre, University College London, Royal Free Campus, London, UK; 7 The Psoriasis Association, Dick Coles House, 2 Queensbridge, Northampton, UK; 8 Institute of Cellular Medicine, Department of Dermatology, Newcastle University, Newcastle upon Tyne, UK; 9 Department of Medical & Molecular Genetics, King’s College London, 9th Floor Tower Wing, Guy’s Hospital, Great Maze Pond, London, UK

**Keywords:** Pustular psoriasis, Anakinra, Randomised controlled trial, Adaptive trial, Small population trial

## Abstract

**Background:**

Palmoplantar pustulosis is a rare but painful and debilitating disease. It consistently ranks the highest of all psoriasis phenotypic variants in terms of symptoms and functional impairment. Management of plaque-type psoriasis has been revolutionised in the last 10 years with the advent of biologic therapies, but treatment options for pustular psoriasis remain profoundly limited. On the basis of mechanistic findings which suggest a key pathogenic role for interleukin (IL)-1 in pustular psoriasis, we hypothesise that anakinra (IL-1 blockade) will be an efficacious treatment for pustular psoriasis.

**Methods/design:**

We will conduct a two-stage, adaptive, double-blind, randomised, placebo-controlled trial to test the hypothesis that anakinra, self-administered daily by subcutaneous injection over 8 weeks, will deliver therapeutic benefit in palmoplantar pustular psoriasis, a localised form of pustular psoriasis typically involving the palms and/or soles. Safety outcomes will be collected for 20 weeks. A total of 64 participants will be randomised to anakinra or placebo in a 1:1 ratio. At the end of stage 1, a decision to progress to stage 2 will be made. This decision will take place after 24 participants have been randomised and followed for 8 weeks and will be based on the ordering of the observed mean outcome values in both treatment arms. At the end of stage 1, the reliability of outcome measurements and method to collect the data will also be assessed, and the primary outcome will be confirmed for stage 2.

**Discussion:**

We have undertaken an adaptive approach in which we will gain proof-of-concept data prior to completing a powered efficacy trial because pustular psoriasis is a rare disease, no validated outcome measures to detect change exist, and limited safety data for anakinra exist in this population. To our knowledge, this will be the first randomised controlled trial that will provide valuable evidence for the efficacy and safety of IL-1 blockade for treatment in pustular psoriasis.

**Trial registration:**

ISRCTN13127147. Registered on 1st August 2016.

EudraCT, 2015-003600-23. Registered on 1st April 2016.

**Electronic supplementary material:**

The online version of this article (10.1186/s13063-018-2841-y) contains supplementary material, which is available to authorized users.

## Background

Pustular psoriasis is characterised by painful, intensely inflamed red skin studded by sheets of monomorphic, sterile, neutrophilic pustules. It may be generalised (generalised pustular psoriasis [GPP]) or localised to the palms and/or soles (palmoplantar pustulosis [PPP]) or nail apparatus (Acrodermatitis continua of Hallopeau) [1]. It consistently ranks the highest of all psoriasis phenotypic variants in terms of symptoms [2] and functional impairment [3], so that the consequent impact is great and equivalent to major medical and psychiatric illnesses [2, 4]. Management of plaque-type psoriasis has been revolutionised in the last 10 years with the advent of biologic therapies driven in great part by the scientific discovery of underlying genetic and immunological disease pathways [5]. In contrast, treatment options for pustular psoriasis are profoundly limited. Other than one small randomised controlled trial (RCT) involving ustekinumab in PPP (*n* = 33) [6], no relevant interventional trials have been published since 2001 [7]. Topical therapy is useful for a minority of cases with mild disease. A Cochrane review of interventions for PPP [8] found evidence for the use of systemic retinoids, a drug class with unpleasant dose-limiting mucocutaneous side effects in most people and of teratogenic potential. The authors of the review also found benefit for oral psoralen and ultraviolet A (PUVA) therapy, an intervention for short-term use that necessitates concomitant oral or topical psoralen and twice-weekly attendance for treatment and which carries a risk of skin cancer. Ciclosporin is only to be ‘considered’ [7], owing to a paucity of evidence, and should not be used beyond 1 year, owing to nephrotoxicity. Tumour necrosis factor (TNF) antagonists, used to great benefit in chronic plaque psoriasis, are largely ineffective [9]. There is thus a significant unmet need for effective treatments with acceptable safety profiles for this patient group.

The poor response in pustular psoriasis to therapies used to great effect in plaque-type disease may be explained by recent evidence indicating that molecular pathways underlying pustular psoriasis are distinct and involve the interleukin (IL)-36/IL-1 axis. Functionally relevant *IL36RN* mutations in both GPP and localised forms have been identified [10–12]. *IL36RN* encodes the IL-36 receptor antagonist (IL-36Ra), an IL-1 family member that antagonises the pro-inflammatory activity of IL-36 cytokines. Disease mutations disrupt the inhibitory function of IL-36Ra, causing enhanced production of downstream inflammatory cytokines, including IL-1 [11, 12]. In keeping with these findings, patients with *IL36RN* mutations significantly upregulate IL-1 production in response to IL-36 stimulation [11]. Regardless of *IL36RN* mutation status, the peripheral blood mononuclear cells of patients with localised pustular psoriasis over-express at least three genes [13, 14] that are consistently up-regulated in IL-1-mediated conditions. These findings suggest a key pathogenic role for IL-1, a cytokine that is known to sustain the inflammatory responses initiated by skin keratinocytes.

Given the proven therapeutic effect of IL-1 antagonists in the treatment of IL-1-mediated diseases, many of which feature neutrophilic infiltration of the skin, we hypothesise that IL-1 blockade will deliver therapeutic benefit in pustular forms of psoriasis. Early proof-of-concept data support this hypothesis: Anakinra, a highly effective IL-1Ra, produced complete and rapid resolution of pustules within days in patients with generalised [15–17], (*n* = 4, including 3 with and 1 without *IL36RN* mutations) and localised disease [18, 19] (*n* = 3, including 2 without *IL36RN* mutations). In two patients with disease relapse on stopping anakinra, pustules cleared on restarting therapy.

Because existing proof-of-concept data for anakinra are limited, in this trial we will first obtain further evidence for benefit and safety prior to completing a fully powered efficacy trial. The study population will be adults with palmoplantar pustulosis (PPP) as the clinical paradigm for all forms, given that it causes very significant disability in its own right, is the most common form, and features chronic development of pustules. Because there are no validated outcome measures of disease change for pustular psoriasis and existing measures include a subjective component, two ‘candidate’ outcome measures will initially be trialled in four centres prior to expanding to the wider multi-centre study.

We will use a two-stage, adaptive, double-blind, randomised, placebo-controlled trial to test our hypothesis that IL-1 blockade with anakinra will deliver therapeutic benefit in pustular forms of psoriasis. At the end of stage 1, a decision to progress to stage 2 to complete the powered efficacy trial will be made on the basis of ordering of the observed mean outcome values in both treatment arms. At this stage, the reliability of measurements and method to collect the data will also be assessed, and the primary outcome will also be confirmed.

## Methods/design

This protocol has been prepared and reported in accordance with the Standard Protocol Items: Recommendations for Interventional Trials (SPIRIT) guidance [20]. The trial SPIRIT checklist can be viewed in Additional file 1.

### Primary objective

Our primary objective in this trial is to determine the efficacy of anakinra as a treatment for adults with PPP compared with placebo. Using a two-stage adaptive design, we will obtain proof of concept prior to completing a fully powered efficacy trial. At the end of stage 1, a decision to STOP or GO to stage 2 will be made, the primary outcome measure will be verified, and safety outcomes will be assessed. The default primary outcome will be fresh pustule count unless Palmoplantar Pustulosis Psoriasis Area and Severity Index (PPPASI) is assessed to be a more reliable and appropriate measurement.

### Secondary objectives

1. To estimate the treatment effect of anakinra in PPP as indicated by change in disease activity over 8 weeks, adjusted for baseline, compared with placebo using PPPASI or pustule count
2. To estimate the time to response of PPP (defined as a 75% reduction in fresh pustule count) and relapse rate (defined as return to baseline fresh pustule count) with anakinra compared with placebo
3. To estimate the proportion of randomised patients who achieve clearance of PPP with anakinra compared with placebo by 8 weeks
4. To estimate any treatment effect of anakinra in pustular psoriasis at non-acral sites as measured by percentage area of involvement at 8 weeks
5. To estimate any treatment effect of anakinra in plaque-type psoriasis (if present) measured using Psoriasis Area and Severity Index (PASI) at 8 weeks
6. To assess adverse event (AE) data to evaluate the harm profile of anakinra
7. To estimate the impact of anakinra on patients’ symptoms and quality of life
8. To estimate the proportion of randomised patients who find the treatment acceptable or ‘worthwhile’
9. To estimate the proportion of randomised patients who adhere to treatment

### Trial design

**Fig. 1:**
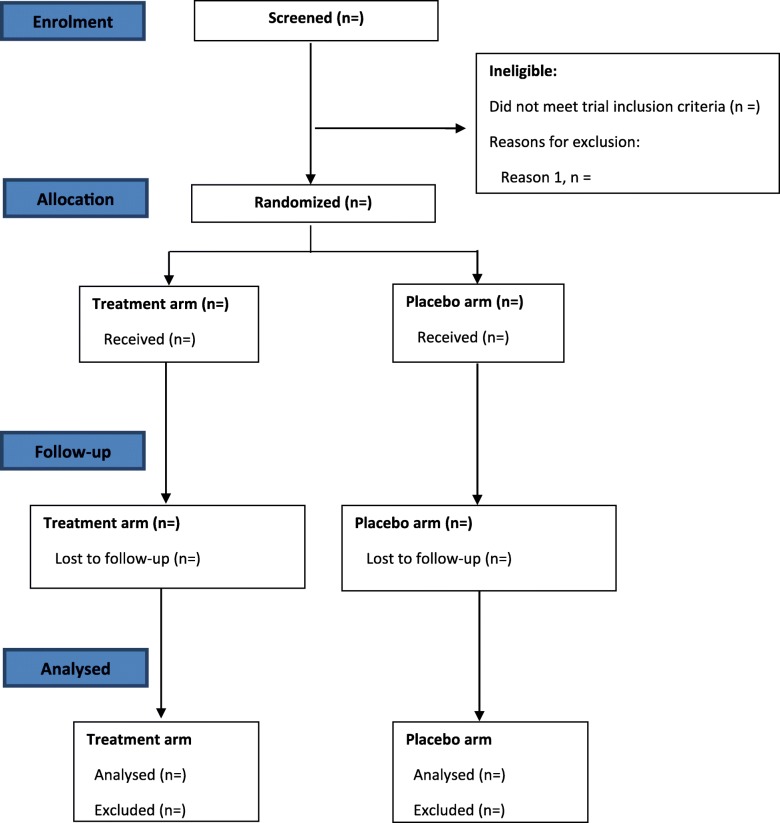
Study flow of participants in APRICOT

**Fig. 2:**
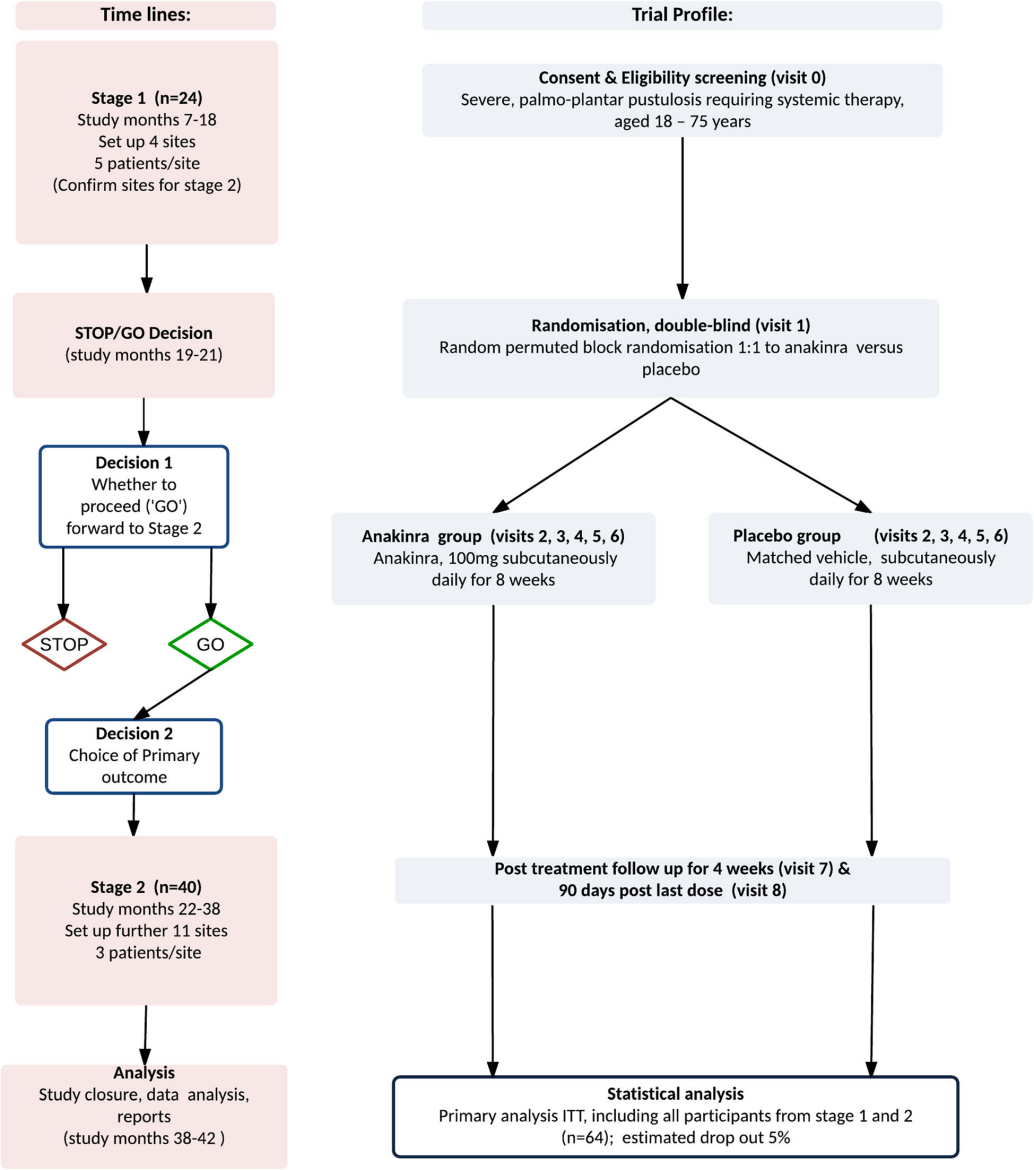
Diagram depicting study design and flow of participants. Decision 1: If placebo does as well or better than treatment arm for both of the two outcomes, Palmoplantar Pustulosis Psoriasis Area Severity Index (PPPASI) or fresh pustule count, the study will STOP. If the treatment arm does better than the placebo arm for at least one outcome the study will proceed (GO). These decisions will be made on the basis of the mean outcome values for each arm. Decision 2: Choice of the primary outcome. If the trial continues (‘GO’), the choice of the primary outcome will by default be the fresh pustule count. If the PPPASI is determined to be more reliable and discriminating than the fresh pustule count then the primary outcome will be PPPASI. *ITT* Intention to treat

APRICOT (Anakinra for pustular psoriasis trial) is a small population, randomised, double-blind, placebo-controlled, multi-centre study with two stages, including a pre-specified adaptive element. The trial will test the superiority of anakinra in PPP. Sixty-four participants will be randomised to either of two parallel arms, as depicted in Fig. 1. Stage 1 will include approximately 4–8 centres (NHS clinics), and the interim analysis will be performed after 24 participants have been randomised and followed for 8 weeks. At this time point, the decision to continue to stage 2, confirmation of the primary outcome and assessment of safety will be made by the independent data monitoring committee (IDMC). The study design can be seen in Fig. 2.

### Decision 1

STOP: Placebo arm does as well as or better than treatment arm for both of the two outcomes; that is, the point estimates are the same, or the point estimate in placebo is less than the treatment arm for fresh pustule count and PPPASI.

GO: Treatment arm does better than placebo arm for at least one measure; that is, the point estimate for the treatment arm is lower than in the placebo arm for at least one of fresh pustule count and PPPASI.

Assuming the GO criteria are achieved, the IDMC will review all safety data from stage 1 and any new drug safety data available through the drug manufacturer and supplier Swedish Orphan Biovitrum (SOBI) or other sources.

### Decision 2

If the outcome of decision 1 is to progress to stage 2, the primary outcome for stage 2 will then be verified. By default, the primary outcome will be fresh pustule count unless PPPASI is assessed to be more reliable and discriminating.

Two statistical analysis plans (SAPs) will be developed. The stage 1 SAP will include a guide to aid the formal decision-making process. The stage 2 SAP will be developed on completion of stage 1 and will detail the full trial analysis. Stage 1 will include approximately 4–6 centres and will take place after 24 participants have been randomised and have completed 8 weeks of follow-up. Stage 2 will include approximately 15–20 centres and a further 40 participants.

### Study population

Our study population will be adults with PPP requiring systemic therapy.

### Inclusion criteria

1. Adults (aged ≥ 18 years) with a diagnosis of PPP made by a trained dermatologist with disease of sufficient impact and severity to require systemic therapy
2. Disease duration > 6 months, not responding to an adequate trial of topical therapy including very potent corticosteroids
3. Evidence of active pustulation on palms and/or soles to ensure sufficient baseline disease activity to detect efficacy
4. At least moderate palmoplantar pustulosis as measured using the Investigator’s Global Assessment (PPP-IGA)

### Exclusion criteria

1. Previous treatment with anakinra or other IL-1 antagonists
2. A history of recurrent bacterial, fungal or viral infections
3. Evidence of active infection or latent tuberculosis or seropositive for human immunodeficiency virus, hepatitis B virus or hepatitis C virus
4. A history of malignancy of any organ system within the past 5 years
5. With moderate renal impairment (CrCl < 50 ml/min), neutropenia (< 1.5 × 10^9^/L) or thrombocytopenia (< 150 × 10^9^/L)
6. Known moderate hepatic disease and/or raised hepatic transaminases (alanine transaminase/aspartate transaminase) more than twice the upper limit of normal at baseline
7. Live vaccinations within 3 months prior to the start of study medication
8. Women who are pregnant, breastfeeding or of childbearing age not on adequate contraception, or men planning conception
9. Poorly controlled diabetes mellitus, cardiovascular disease or asthma, or concomitant therapy that may interact with anakinra
10. Unable to given written informed consent or comply with the study visit schedule

Participation will also be excluded if there is use of therapies with potential or known efficacy in psoriasis during or within the following specified time frames before treatment initiation:

1. Very potent topical corticosteroids within 2 weeks
2. Topical treatment that is likely to impact signs and symptoms of psoriasis within 2 weeks e.g. corticosteroids, vitamin D analogues, calcineurin inhibitors, retinoids, keratolytics, tar, urea
3. Methotrexate, ciclosporin, acitretin, alitretinoin within 4 weeks
4. Phototherapy or PUVA therapy within 4 weeks
5. Etanercept or adalimumab within 4 weeks
6. Infliximab or ustekinumab or secukinumab within 3 months
7. Other TNF antagonists within 3 months
8. Other immunosuppressive or immunomodulatory therapy within 30 days or 5 half-lives prior to treatment initiation, whichever is longer
9. Any other investigational drugs within 30 days (or 3 months for investigational monoclonal antibodies) or 5 half-lives prior to treatment initiation, whichever is longer

### Intervention

The active arm will receive anakinra (Kineret; SOBI, Stockholm, Sweden) 100 mg/0.67 ml daily through self-administered subcutaneous injection. SOBI will supply the investigational medicinal in pre-filled syringes. The control arm will receive identical matched syringes containing 0.67 ml of vehicle solution only. Each patient will self-administer a daily subcutaneous injection of investigational medicinal product (anakinra or placebo) for 8 weeks and will be followed for 12 weeks post-randomisation with a final safety follow-up visit at approximately 20 weeks (90 days after last trial treatment).

### Adherence and concomitant medication

Participants will receive daily text reminder messages to encourage them to comply with the daily dosing schedule and will be asked to respond to the text to confirm that they have taken their medication. Those patients who are unable to provide a mobile phone number for text reminders or do not wish to will be asked at each visit for a record of their daily injections.

Permitted medications include topical therapy such as hydrocortisone, antihistamine for injection site reactions, and mild topical corticosteroids for the treatment of psoriasis at sites other than hands and feet. The use of a potent corticosteroid as ‘rescue’ topical therapy will be dispensed and recorded by the study team. Very potent topical corticosteroids (e.g., clobetasol propionate 0.05%), along with any topical treatment likely to impact signs and symptoms of PPP, are prohibited.

### Outcomes

The primary outcome will be confirmed at the end of stage 1 and will be as follows:

1. Fresh pustule count on palms and soles across 1, 4 and 8 weeks (adjusted for baseline fresh pustule count on palms and soles)

The count will include pustules macroscopically visible, white/yellow in colour with no brown colour, and present on the glabrous skin of the palms and/or soles.

OR

2. PPPASI across 1, 4 and 8 weeks (adjusted for baseline PPPASI)

The PPPASI has been adapted from the PASI by Bhushan et al. [21] and has been used as the primary outcome measure in previous trials evaluating interventions in PPP [6, 8].

#### Secondary outcomes

Investigator-assessed efficacy measures will be as follows:

1. Fresh pustule count on palms and soles OR PPPASI, depending on the primary outcome confirmed at stage 1
2. Total pustule count (pustules must be macroscopically visible, white/yellow/brown in colour, with or without crust) on palms and soles across weeks 1, 4 and 8 adjusted for baseline
3. PPP-IGA at weeks 1, 4 and 8 adjusted for baseline (clear, almost clear, mild, moderate, severe)
4. Time to response of PPP (a 75% reduction in fresh pustule count)
5. Time to relapse (defined as return to baseline fresh pustule count)
6. Time to achievement of ‘clear’ on PPP-IGA by 8 weeks
7. Development of a disease flare (> 50% deterioration in PPPASI)
8. Pustular psoriasis at non-acral sites (not hands and feet) as measured by percentage area of involvement at 8 weeks
9. Plaque-type psoriasis (if present) measured using PASI at 8 weeks

Safety measures will include the following:

- Serious infection as defined by any infection leading to death, hospital admission or requiring intravenous antibiotics
- Neutropenia (neutrophil count of ≤ 1.0 × 10^9^/L)
- All reported AEs, adverse reactions (ARs), unexpected adverse reactions (UARs) and serious AEs, ARs and UARs

Patient-reported efficacy outcomes are as follows:

- Patient’s global assessment (clear, nearly clear, mild, moderate, severe, very severe) across 1, 4 and 8 weeks
- Palmoplantar Quality of Life Instrument score at 8 weeks [3]
- Dermatology Quality of Life Index at 8 weeks [22]
- EQ-5D-3L score at 8 weeks [23]
- Treatment acceptability (five questions, such as whether the treatment is ‘worthwhile’) evaluated using a brief questionnaire with a response scale of 1–5 at week 12
- Adherence to treatment as measured by responses to daily text messages over 8 weeks

**Fig. 3:**
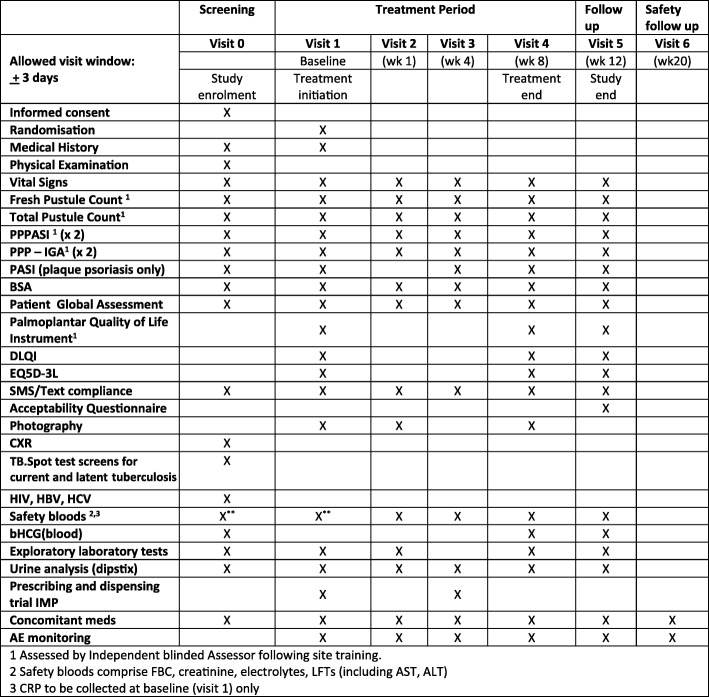
Study procedures of APRICOT. *AE* Adverse event, *bHCG* Beta human chorionic gonadotropin, *BSA* Body surface area, *CXR* Chest x-ray, *DLQI* Dermatology Quality of Life Index, *EQ-5D-3L* Three-level version of EQ-5D instrument, *HBV* Hepatitis B virus, *HCV* Hepatitis C virus, *HIV* Human immunodeficiency virus, *IMP* Investigational medicinal product, *PASI* Psoriasis Area and Severity Index, *PPPASI* Palmoplantar Pustulosis Psoriasis Area and Severity Index, *PPP-IGA* Palmoplantar pustulosis as measured using the Investigator’s Global Assessment

The schedule of trial enrolment, interventions and assessments is presented in Fig. 3. Primary outcome assessments of fresh pustule count and PPPASI will be carried out by an independent assessor blind to study treatment at each site. During stage 1, a second assessor, blind to treatment, will also assess PPPASI and PPP-IGA at each site, and photography will be completed to enable a central blinded assessor to evaluate fresh pustule count to inform decision 2 (primary outcome for stage 2).

## Statistical considerations

### Randomisation and blinding

Participants will be randomised to treatment in a 1:1 allocation ratio using blocked randomisation stratified by centre via an online system to ensure allocation concealment. The study team, treating clinicians, independent outcome assessors and participants will be blind to treatment arm. The study statistician will be subgroup-blind (analysing arms labelled as ‘A’ and ‘B’) and will not be involved in the analysis at stage 1. The analysis at stage 1 will be undertaken by the second statistician, who will be subgroup-blind during the analysis but unblinded at data review. The IDMC will operate unblinded. A 24-hour code break and medical information system will be used to unblind healthcare workers in case of an emergency. The chief and principal investigators will be told of incidents, and the trial statistician will be informed at the analysis stage of the trial.

### Sample size

Because the potential primary outcome at this stage is unknown, the sample size has been calculated by reference to a standardised effect size. An effect size of 0.9 SD was chosen with consideration of the cost of the drug and motivation for patients to adhere to treatment, given the requirement for daily self-administered subcutaneous injections. In addition, larger effect sizes have been reported with oral retinoids [8, 24], a recommended systemic intervention for pustular psoriasis. To detect a difference of 0.9 SD with power 90% using a 5% significance level, a sample size of 27 per arm would be required. RCTs involving placebo arms [21, 8] have observed withdrawal rates of < 5%. We aim to recruit 32 participants per arm (*N* = 64 in total), which will allow for an approximate 15% withdrawal rate.

#### Stage 1 sample size

The sample size for stage 1 is based on correct ordering of group means. We want a high probability of continuing (‘GO’) if there is a true (conservative) difference in means between the groups of 0.5 SD, in favour of the treatment group. With 20 patients (*n* = 10 per arm), assuming a real difference of 0.5 SD, the probability that the means for treatment arms will be correctly ordered (i.e., treatment mean greater than placebo mean) is 0.85. If two outcomes are assessed, each with an expected difference of 0.5 SD, then the overall probability of failing to GO is (1 − 0.85)^2^ = 0.0225 (i.e., less than 3 in 100). There is thus a minimal chance of failing to continue if the treatment really is beneficial. If there is no treatment benefit, the probability of not progressing to the next stage is 0.25 based solely on these rules. Whilst this is low, the balance of errors has been selected to allow optimal identification of treatment benefit and at most could only be 0.5 under this design. Stage 1 does not involve statistical tests. To ensure that 10 participants contribute to each arm, the interim analysis for stage 1 will occur after 24 participants have been randomised and followed.

### Analysis

All analyses will be based on the intention-to-treat principle and will include all participants in the treatment arms to which they were allocated, regardless of treatment subsequently received. A detailed SAP will be written for stages 1 and 2 separately. The stage 1 SAP will detail the analysis to assess the reliability and discriminative ability of the two proposed primary outcomes. The decision to continue to stage 2 will occur if the point estimates for the mean fresh pustule count OR mean PPPASI are greater in the active arm than in the placebo arm. The point estimate for the mean will be the baseline adjusted treatment group differences (averaged over 1, 4 and 8 weeks for each patient), calculated using linear regression. To guide the decisions at the end of stage 1, a number of different descriptive analyses will be performed: Outcome distributions will be plotted; the standardised mean difference will be calculated by time point; agreement between the ‘site’ assessors and the central ‘photographic’ assessment will be assessed using the method of Bland and Altman [25], allowing for the multiple observations; and the intraclass correlation coefficient will be calculated using a mixed effects analysis of variance. Stage 1 will also include a full review of all safety data. These data will be presented in line with the prior IDMC reports and will predominantly include AEs tabulated by treatment arm coded at the preferred term level for reporting. When useful, time-to-event analyses will be undertaken to examine the difference in time-to-event curves between the two arms, and the hazard function will be plotted to assess consistency in risk over time. No hypothesis testing will be undertaken for AE outcomes.

At the end of stage 2, the treatment effect will be estimated using a linear (Gaussian) mixed model on weeks 1, 4 and 8 data. Participant will be included as a random intercept, with fixed effects for time, time-by-treatment group interaction and baseline score of the primary outcome. Centre will be included in the model as either a random or fixed effect, depending on the total number of centres recruiting to the study and the average number of participants recruited from each centre. The estimated treatment effect at 8 weeks will be reported with a 95% CI and corresponding *p* value as the primary outcome. We will also report the treatment effect at weeks 1 and 4. The fresh pustule count and PPPASI profiles will be plotted over time for individuals to examine longitudinal patterns and help determine the necessity of including a random intercept term in the model. A sensitivity analysis will then be undertaken for the primary analysis with adjustment for use of rescue medication. The proportion of participants using rescue medication and the amount used will be summarised by treatment arm. The primary analysis will be repeated, including 12-week follow-up data. All treatment effect estimates will be reported with 95% CIs, and a 5% significance level will be used for the primary outcome test. Every effort will be made to obtain follow-up data for all participants, including those who stop treatment. The analytical methods described above will employ maximum likelihood estimation and thus are efficient for handling missing outcome data under a missing at random (MAR) assumption. When required, sensitivity analyses will investigate the robustness of the results to the MAR assumption.

Continuous secondary outcomes will be analysed using the same modelling approach as specified above. Binary outcome data will be analysed using logistic regression models. Kaplan-Meier curves will be plotted for time to response and time to relapse. A complementary log-log model will be fitted to estimate the treatment effect for the time-to-event outcomes. If the proportional hazards assumption is not met, an alternative parameterisation will be used or an alternative time-to-event model will be sought.

### Data management

Data will be managed using the MACRO database system (InferMed, London, UK). This system is regulatory compliant and will be maintained by the King’s Clinical Trials Unit. It will be hosted on a dedicated secure server within King’s College London. The quality assurance manager (sponsor) will conduct internal audits to check on compliance with International Conference on Harmonisation of Technical Requirements for Registration of Pharmaceuticals for Human Use guidelines for good clinical practice (GCP), meeting the requirements of the Medicines and Healthcare products Regulatory Agency (MHRA). The audits will also include laboratory activities according to an agreed audit schedule, taking into consideration the 2009 MHRA guidelines for GCP in the laboratory.

### Trial oversight committees

The IDMC will be responsible for monitoring evidence for treatment harm and reviewing all decisions made in relation to the safety aspects of the study. The IDMC will meet on initiation of the project and agree on the type and frequency of meetings. They will review all data at completion of stage 1 and advise on the decision to stop or continue the trial based on the pre-specified criteria and any emerging safety concerns. They will advise on the primary outcome for the trial using the SAP for stage 1 to guide their decision making.

The trial steering committee (TSC) will include an independent chair, two independent members, an independent patient representative, the chief investigator and at least one study statistician. The TSC will meet as required with invited observers from the Efficacy and Mechanism Evaluation (EME) programme. The TSC is the main decision-making body. It will have overall responsibility for scientific strategy and direction and has ultimate responsibility for ensuring that the project’s aims are delivered on time and within budget. Specific roles, meeting frequency and timelines will be detailed in the TSC Terms of Reference.

## Discussion

The treatment options for PPP are limited, and this trial will provide evidence on an efficacy and safety profile for anakinra for short-term treatment (8-week treatment period and efficacy evaluation with further efficacy data collected at 12 weeks and safety data up to 20 weeks). We were unable to take a conventional approach to the design of the study, owing to several constraints: PPP is a rare disease; limited safety data for anakinra exist; and there is no validated outcome for PPP. Rather than not undertake trials in small populations when it is not possible to use traditional approaches, there has been a call to change our approach to and thinking about such studies [26]. The size of the study means this trial will be able to detect a benefit only if the true effect size is fairly large. The limitation of not detecting a smaller effect size was made with consideration of the trade-off between the minimum benefit felt necessary with the requirement for daily self-administered subcutaneous injections along with the cost of the drug, as well as existing effect sizes observed in drug treatments in this disease [8, 24]. This decision was made with input from clinicians and patients. In addition to the limited sample size owing to the small population, we also faced three other obstacles: uncertainty around a suitable outcome measure that could capture change in PPP disease, lack of proof-of-concept data, and minimal safety information for this drug in this population. Because we are extremely limited by the number of total participants we can recruit, this led us to address these uncertainties in the current trial by incorporating an interim trial with the potential to stop the trial after 24 patients if the results flag concern for safety or if there is no signal for efficacy. We were unable to use a conventional approach to the design of the interim stage because this would result in a prohibitively large sample size. It was deemed reasonable to accept the higher-than-desired probability for progressing if there is no true benefit in light of the overall total sample size along with knowledge that the trial would be closely monitored and stopped for safety if required. This study includes a parallel mechanistic evaluation, so this study will allow collection of relevant material designed to investigate underlying disease pathogenesis which will be informative for future treatment development and symptom control.

## Trial status

The APRICOT trial received ethical approval on 1st April 2016. The first participant was enrolled on 21st September 2016, and the trial in still recruiting participants for stage 1 and has randomised 31 participants.

## Additional file

Additional file 1: SPIRIT 2013 checklist: recommended items to address in a clinical trial protocol and related documents. (DOCX 42 kb)

## Abbreviations

AE: Adverse event
APRICOT: Anakinra for pustular psoriasis trial
AR: Adverse reaction
DLQI: Dermatology Quality of Life Index
EME: Efficacy and Mechanism Evaluation programme
GCP: Good clinical practice
GPP: Generalised pustular psoriasis
IDMC: Independent data monitoring committee
IL: Interleukin
IL-36Ra: Interleukin-36 receptor antagonist
MAR: Missing at random
MHRA: Medicines and Healthcare products Regulatory Agency
NIHR: National Institute for Health Research
PASI: Psoriasis Area and Severity Index
PPP: Palmoplantar pustulosis
PPPASI: Palmoplantar Pustulosis Psoriasis Area and Severity Index
PPP-IGA: Palmoplantar pustulosis as measured using the Investigator’s Global Assessment
PUVA: Psoralen and ultraviolet A therapy
RCT: Randomised controlled trial
SAP: Statistical analysis plan
SPIRIT: Standard Protocol Items: Recommendations for Interventional Trials
TNF: Tumour necrosis factor
TSC: Trial steering committee
UAR: Unexpected adverse reaction
